# Antioxidant Synergy in a Mixture of Powder Plant Leaves and Effects on Metabolic Profile, Oxidative Status and Intestinal Morpho-Histochemical Features of Laying Hens

**DOI:** 10.3390/ani15030308

**Published:** 2025-01-22

**Authors:** Angela Gabriella D’Alessandro, Alessio Di Luca, Salvatore Desantis, Giovanni Martemucci

**Affiliations:** 1 Department of Soil, Plant and Food Sciences (DiSSPA), University of Bari Aldo Moro, 70126 Bari, Italy; angelagabriella.dalessandro@uniba.it (A.G.D.); alessio.diluca@uniba.it (A.D.L.); gmartem@libero.it (G.M.); 2 Department of Precision and Regenerative Medicine and Ionian Area (DiMePRe-J), University of Bari Aldo Moro, 70124 Bari, Italy

**Keywords:** olive, rosemary, laurel, antioxidant activity, synergism, diet leaf mixture, biochemical parameters, gut histomorphology, histochemistry, laying hens

## Abstract

Phenolic antioxidant intake is encouraged to prevent oxidative damage, and antioxidant synergy is considered an advantage in adding polyphenols from varied plants. This study investigated the antioxidant and synergistic interactions among olive leaf (OL), bay laurel (BL), and rosemary (RL) leaf powder mixture (LPM: OL + BL + RL), using in vitro chemical tests [TPC, ORAC, TEAC-ABTS, FRAP; combination index (CI)], and in vivo validation on blood oxidative status, metabolic profile, and intestinal histomorphology in laying hens. The in vitro study indicated a whole higher antioxidant capacity for the LPM than respective single/double-leave combinations. The LPM CI value (IC50, 0.60) indicated a synergistic effect compared to the binary combinations. Thus, the LPM was validated in vivo through dietary supplementation on sixty Lohmann Brown hens (30 weeks old), reared in an indoor–outdoor rearing system divided. The hens were allocated into two experimental groups (n. 30): basal control diet group; and diet supplemented group with 6 g/kg feed of LPM) containing OL, BL, and RL (respectively, at 65.7%:18.9%:15.4%), for 60 days. The LPM improved (*p* < 0.05) the oxidative status (TAS, FRAP; ROMs, TBARs) and vitamin E level, metabolic and immunological profiles, and it induced region-specific changes in the morphology and carbohydrate composition of mucins along intestinal tracts of the animals. These findings could provide a valuable strategy for identifying synergistic combinations in functional feed formulations for laying hens.

## Simple Summary

This study investigated the antioxidant synergism activity of combining three plant-based antioxidants, olive, bay laurel, and rosemary leaves into a single dietary supplement for laying hens. These plants are rich in phenolic compounds, which help prevent oxidative damage, a process that can harm cells and tissues. The researchers compared the in vitro antioxidant effects of the individual leaves, and in binary (in pairs), and in a ternary combination of all three leaves (termed LPM). Among the individual leaves, olive leaves showed higher antioxidant capability; the ternary LPM displayed synergistic activity. In an in vivo study on hens, the LPM feeding supplement improved oxidative markers, increased vitamin E levels in the blood, supported better liver and immune function, and improved intestinal health by enhancing nutrient absorption structures. The findings suggest that the mixture of olive, rosemary and laurel leaves have a synergistic effect as antioxidant benefits, potentially leading to healthier poultry and possibly enhancing the quality of products for consumers.

## 1. Introduction

Free radicals, which cause oxidative stress, contribute to various pathological conditions in humans [1,2] and animals [3]. Several stress factors associated with poultry production may compromise the health and productivity of laying hens and broilers [4,5].

Phytogenic feed supplements, such as intact herbs, spices, and their extracts, can positively impact poultry health and performance, largely due to their polyphenol content [6,7]. Polyphenols provide beneficial effects in poultry nutrition and performance by enhancing antioxidant status, metabolic profile [5,8,9], and promoting a healthy gastrointestinal tract [10,11].

The high levels of antioxidant compounds in the leaves of *Olea europaea* L. (*O. europaea*), *Laurel nobilis* L. (*L. nobilis*), and *Rosmarinus officinalis* L. (*R. officinalis*), along with their antioxidant properties [12,13,14], make these plants promising for combating harmful substances linked to various stressors in poultry production.

Olive leaves (from *O. europaea* tree; Oleaceae family) and their extracts positively affect performance in hens [15,16] and broilers [17,18]. Bay laurel (*L. nobilis*) is an aromatic and medicinal plant, which belongs to the Laureacea family, and it is characterised by anti-inflammatory and antioxidant activities [12,19]. Rosemary (*R. officinalis* L.), a herb from the Labiatae family, has biological activities [20], including antioxidant, anti-inflammatory, and immunomodulatory properties [9,14,21].

Dietary supplementation with multifunctional antioxidants can offer superior protection compared to single antioxidant compounds [22]. For instance, a diet supplemented with 14 herbs improved feed efficiency and body weight gain in broilers [23]. Studying how antioxidant properties are modulated by the proportions of plant products in mixtures used in diets is pivotal. The antioxidant capacity of herbs and vegetable mixtures is attributed to the synergistic, antagonistic, or additive effect of their diverse phytochemicals [24,25,26]. Combination treatments have gained increasing importance in recent years, aiming to achieve greater efficacy, focusing on the benefits of the possible synergism [27], as this could optimise the blend component.

Different assays are available to evaluate the antioxidant capacity of substances, with the Folin–Ciocalteu (FC) assay for measuring total phenolic content (TPC), ORAC, FRAP, and TEAC-ABTS among the most commonly used. These assays operate on two possible chemical mechanisms, such as hydrogen atom transfer (HAT; ORAC) or single electron transfer (SET; FC, FRAP), while TEAC-ABTS combines both in a mixed mode mechanism (HAT—SET) [28,29]. Due to the specificity and sensitivity of each method, a more accurate evaluation of antioxidant activity can be achieved by combining several assays [30]. For the quantitative determination of drug/compounds interactions, the Chou–Talalay method [31] has gained popularity over the past 20 years. This method is based on the combination index (CI), which indicates synergistic, additive or antagonistic effects. CompuSyn software allows the determination of these relations by automated simulations [32].

It is also important to investigate whether the health benefits of phenolic compounds in food can be linked to antioxidant activity both in vitro and in vivo. The aims of this study were to evaluate (a) the in vitro antioxidant activities of individual leaves (olive, bay and rosemary) and their binary and ternary mixtures; (b) the interaction as synergistic, additive, or antagonistic effects in the obtained mixtures; and (c) the in vivo validation in laying hens of the combination identified through chemical-based antioxidant and interaction assessments.

## 2. Materials and Methods

### 2.1. Plant Material and Sample Preparation

Olive leaves (*O. europaea*) were harvested during the autumn (mid-September to October) pruning from the olive trees representing the most common cultivars of the Apulia region in southern Italy, including ‘Coratina’, ‘Cima di Bitonto’, ‘Pizzutella Barese’, and ‘Bambina’. These leaves were then processed to obtain a sample with an equal amount from each cultivar. Rosemary (*R. officinalis*) and bay laurel (*L. nobilis.*) leaves were collected from the same geographical area in the summer. All harvested leaves were air-dried at room temperature for two days, followed by drying at 40 °C for 48 h (AOAC, 2000; 7.045 method). The dried leaves were then ground into a fine powder using a cutting mill equipped with rotating knives and a 1 mm sieve. Powder samples were prepared individually, as well as in binary (1:1 ratio; olive and rosemary; olive and laurel; rosemary and laurel) and ternary (1:1:1 ratio; olive, rosemary and laurel) combinations. The individual leaf powders—olive leaf (OL), bay laurel (BL), and rosemary (RL)—and their mixtures as binary (in pairs) and ternary (LPM) combinations were stored at room temperature and in low humidity and kept in the dark until analysis.

### 2.2. In Vitro Antioxidant Capacity Evaluation

The antioxidant activities of individual leaves (OL, BL, and RL) and their binary (OL + BL, OL + RL, BL + RL) and ternary (OL + BL + RL) mixtures were determined by total phenolic content (TPC), ferric reducing antioxidant power (FRAP), oxygen radical absorbance capacity (ORAC) and trolox equivalent antioxidant capacity (TEAC-ABTS). All the assays were performed in triplicate.

#### 2.2.1. Chemicals

All the chemicals were of analytical grade and were purchased from Sigma–Aldrich (Milan, Italy), and were 2,2′-azobis(2-amidinopropane) dihydrochloride (AAPH), potassium persulfate, 6-hydroxy-2,5,7,8-tetramethylchroman-2-carboxylic acid (Trolox), 2,2-Azino-bis-(3-ethylbenzothiazolin-6-sulfonic acid) diammonium salt (ABTS), Folin–Ciocalteu reagent, gallic acid, ultra-pure water, ethanol, sodium acetate, acetic acid, hydrochloric acid, 2,4,6-tri(2-pyridyl)-1,3,5-triazine (TPTZ), iron(III) chloride (FeCl3), phosphate-buffer saline (PBS), and potassium persulfate (K2S2O8).

Extraction of the lipophilic compounds from leaf powder samples was carried out in ethanol (80%; 1:10, *w*/*v*) for 15 min using an ultrasonic cleaner (CP102; FIOA International s.r.l., Arezzo, Italy). The extract was filtered through a 0.45-µm PTFE syringe filter (CH4525-NPL, Fisher Scientific Italia, Rodano, Milano, Italy) and stored at −20 °C. All procedures were conducted in the dark to avoid oxidation. The lipophilic extracts were used to assess TPC, TEAC-ABTS, ORAC, and FRAP.

#### 2.2.2. TPC Determination

The total phenolic content (TPC) of the dried leaf powders was assayed spectrophotometrically using a modified Folin–Ciocalteu method [33]. Briefly, 2.5 mL Folin–Ciocalteu reagent, 2 mL of 7.5% aqueous sodium carbonate solution, and 0.5 mL of lipophilic extract were mixed well. After 90 min of storage at room temperature (RT) (20–25 °C) in the dark, the absorbance of the mixture was read at 765 nm with a UV–Visible spectrophotometer (PerkinElmer Lambda 15; PerkinElmer Italia Spa, Milano, Italy). A mixture of solvent and reagents was used as a blank matrix. The TPC was expressed as milligramme of gallic acid equivalents (GAE) in grammes of dry weight, using a gallic acid standard curve (0–250 mg/L).

#### 2.2.3. ORAC Assay

The hydrophilic antioxidant capacity was measured using the ORAC method as described by Huang et al. [34]. Briefly, dilution of each sample extract and Trolox calibration solutions were prepared in phosphate buffer, pH 7.4, at the specified final concentrations. Sample (20 µL) and fluorescein solution (160 µL, 8.16 × 10^−5^ mM) were added to a 96-well microplate (Thermo Scientific Fisher Italia, Rodano, Milano, Italy), and the mixture was incubated at 37 °C for 20 min, followed by addition of 20 µL of AAPH solution (153 mM). Fluorescence was monitored at 493 and 515 nm (excitation and emission wavelengths, respectively) at 1 min intervals for 50 min with a microplate reader (Tecan Infinite^®^ 200 PRO; Männedorf, Switzerland). A calibration curve was prepared with different concentrations of Trolox (5–50 μM). The effects were expressed as µmol Trolox equivalents (TEs) in grammes of dry weight sample (µmol TE/g dw).

#### 2.2.4. FRAP Assay

The antioxidant activity was measured using the FRAP assay according to Benzie et al. [35] with minor modifications [36]. Each sample extract, opportunely diluted, along with Trolox calibration solutions, was prepared in distilled water. FRAP reagent was prepared by mixing 20 mL of acetate buffer (pH 3.6), 2 mL of 2,4,6-tripyridyl-s-triazine (TPTZ) solution (10 mM, dissolved in 400 mM HCl), and 2 mL of iron (III) chloride (FeCl3.6H2O) solution (20 mM). Aliquots (25 µL) of the different sample extracts and FRAP reagent (250 µL) were added in a 96-well microplate and incubated at RT for 8 min in the dark. Absorbance readings were taken at 593 nm, and a calibration curve was prepared using varying concentrations of Trolox (0.1–1 mM). Results were expressed as µmol of Trolox equivalents (TEs) in grammes of dry weight sample (µmol TE/g dw).

#### 2.2.5. TEAC-ABTS Assay

Radical cation scavenging capacity against ABTS + radical (2,2′ -azino-bis(3-ethylbenzothiazoline-6-sulfonic acid), was determined using the method described by Re et al. [37]. The green ABTS radical cation (ABTS•+) was first produced by incubating ABTS stock solution (7 mM) with 2.45 mM potassium persulfate for 12–24 h at RT in the dark. Before the measurements, the ABTS• solution was diluted (approximately 1:80) with ethanol (80%) to obtain an absorbance of 0.700 ± 0.02 at 734 nm. For the photometric assay, 3 mL of the ABTS•+ solution and 30 μL of extract solution from leaves (diluted 1:10) were mixed for 45 s and stood for 6 min at RT (20–25 °C) before the absorbance was measured at 734 nm (UV–visible spectrophotometer Perkin–Elmer Lambda 15). A calibration curve was prepared with different concentrations of Trolox (1–4 mM). The ABTS radical scavenging capacity was expressed as mM Trolox equivalents (TEs) in grammes of dry weight sample (mM TE/g dw).

#### 2.2.6. Combination Index (CI) Analysis

To analyse the synergy among the components of LPM, potential binary and ternary antioxidant interactions between OL, BL, and RL were evaluated using the combination index (CI) method of Chou–Talalay [31,38]. The determination of CI and fraction affected (Fa)-CI simulation was developed by CompuSyn software 1.0. (https://compusyn.software.informer.com/1.0/, accessed on 19 January 2025), using the ORAC antioxidant assay values.

The CI was used to describe the effects of combination as synergistic, additive, or antagonistic based on the following values: less than 0.3, CI exhibit strong synergism, whereas value of 0.3–0.69 possess synergism; 0.70–0.84, 0.85–0.89 shows moderate synergism and mild synergism, respectively; 0.9–1.09 exhibit additive effect; 1.10–1.19 indicates slight antagonism; 1.20–1.44 possess antagonism; and >1.45 indicates moderate to strong antagonism [39].

On the basis of the best LPM results in vitro, the ternary LPM combination was assessed in an in vivo study.

### 2.3. In Vivo Study

To validate the in vivo efficiency of the ternary LPM (OL + OR + BL) combination, a test was conducted in laying hens to evaluate its dietary effects on blood metabolic profile, immunological parameters, oxidative status, intestinal histomorphology, and histochemical characteristics.

#### 2.3.1. Animal Management and Diets

Experimental protocol and animal care procedures were approved by the Institutional Ethics Committee of the Department of Emergencies and Organ Transplantation of the University of Bari (Prot n.04/2020). The trial lasted 60 days and was carried out in autumn, in a private farm located in South Italy, Apulia region (latitude 41 04’ N, 17 05’ E, 5 m s.l.m.), on 60 Lohman Brown laying hens, (30 weeks old) with a body weight of 1800 ± 130 g. All hens were vaccinated according to the vaccination schedule required by commercial systems. Weight was used to divide the hens into two equally sized groups (n.30) that corresponded with the dietary treatments. The dietary treatments included a commercial basal diet as control (CON) and an experimental diet as a commercial diet supplemented with LPM at a dosage of 6 g/kg feed, selected on the basis of a careful literature search. Based on in vitro results related to antioxidant interaction activity, the individual leaf content in the LPM was established as follows: OL, 65.7%; RL, 18.9% and BL, 15.4%, corresponding to 3.94, 1.13, 0.93 g/Kg feed for OL, RL and BL, respectively. The feed for laying hens was prepared according to the nutritional requirements reported by NRC (1994), and the nutrition levels are shown in Table 1.

Given that a rearing system with access to an open area offers high welfare potential for laying hens [40,41], the hens were reared in an indoor–outdoor rearing system with two pens (4 m^2^/hen), one for each experimental group, separated by a 2 m high net to prevent mixing. The surface of the outdoor area consisted of soil without plant cover. Each enclosure contained a poultry house at a low density (6 hens/m^2^) with nests (1/6 hens) and perches, allowing the hens free access to the outdoor enclosure (outside option from 08:00 h to 18:00 h). Feed and water were offered ad libitum. A photoperiod of 16 h light and 8 h dark was maintained with artificial light.

#### 2.3.2. Blood Sampling and Laboratory Analyses

At the conclusion of the experiment, from the brachial vein of 30 hens randomly selected from each experimental group, approximately 2 mL of blood samples were collected in plastic vacuum tubes (BD Vacuum Advance in Becton Dickinson, Franklin Lakes, NJ, USA). The serum (after clotting) and plasma were separated by centrifugation at 3000 rpm (1814× *g*) for 15 min, and the aliquots transferred into plastic vials were stored at −20 °C until the analysis of biochemical, immunological and antioxidant parameters. Blood biochemical characteristics were determined using an automated biochemical analyser (TC-220 TECOM, Jiangxi, China) using commercial kits according to the colourimetric method.

The serum concentration of triglycerides (TG, monitored at 505 nm), total cholesterol (TC, monitored at 505 nm), high-density lipoprotein-cholesterol (HDL-c, monitored at 570 nm), low-density lipoprotein-cholesterol (LDL-c), alkaline phosphatase (ALP, monitored at 405 nm), aspartate aminotransferase (AST), were determined using diagnostic kits by SPINREACT (Sant Esteve de Bas, Girona, Spain). Alanine aminotransferase (ALT monitored at 340 nm) was estimated using a commercial kit by PRO-EKO (Petacciato, Campobasso, Italy).

The inflammatory cytokines TNFα, IL-1β, and IL-6 were measured using the test kits provided by Immunological Sciences (Rome, Italy) following the manufacturer’s instructions, using an infinite TECAN M1000Pro plate reader (Tecan, Mannedorf, Switzerland).

The oxidative status of the animals was evaluated by the assessment of the following: total antioxidant status (TAS), FRAP, ROMs, and TBARs. Measurements of TAS were performed spectrophotometrically according to [42], and the results were expressed as Trolox equivalent/L units. Plasma samples were used for reactive oxygen metabolites (ROMs), and thiobarbituric reactive substances (TBARSs). ROM values were determined through a spectrophotometer and a colourimetric method using a commercial kit (Diacron, Grosseto, Italy) according to the manufacturer’s instruction, at a wavelength of 505 nm [43]. Results were expressed in Carr units (1 U/Carr corresponds to 0.024 mmol/L of H_2_O_2_). The determination of TBARSs was performed by spectrophotometric reading according to the [44], using a standard curve with 1,1,3,3 tetramethoxypropane (Sigma Aldrich, St. Louis, MO, USA). Trichloroacetic acid (10%, *v*/*v*) was added to the plasma samples, promoting the precipitation of proteins; the resulting mixture was incubated on ice for 15 min. After centrifugation at 2200 rpm at 4 °C for 15 min, 0.67% thiobarbituric acid (Sigma Aldrich, St. Louis, MO, USA) was added to the supernatant, which was then incubated in a water bath at 90 °C for 10 min and the absorbance was read at 532 nm in a spectrophotometer. The results were expressed as micromoles of MDA (malondialdehyde) per litre of plasma. The FRAP test, according to [35], evaluates the antioxidant activity of plasma by reducing the ferric iron complex in an acidic environment. The results were expressed in mM/mL.

Vitamin A and E concentrations were analysed through the HPLC method as described by Zhao et al. [45]. Briefly, retinol and alpha-tocopherol were extracted from plasma samples with chloroform and analysed on an HPLC (Kontron Instruments, Italy) system constituted of an autosampler (HPLC Autosampler 360) with a 20 µL loop, a high-pressure mixing pump (HPLC Pump 422) and a C18 column (5 µm, 250 mm, 4,60 mm) (Phenomenex, Torrance, CA, USA). The mobile phase was 100% methanol at a flow rate of 1.0 mL/min. A fluorometer detector (SFM) with two different wavelengths (Vitamin A at 325 nm, Vitamin E at 300 nm) and a computer with Kroma System 2000 software were used. The concentrations of vitamins were determined using retinyl acetate and α-tocopherol acetate as internal standard and the time of elution of pure standards. Results were expressed as µg/mL of plasma.

#### 2.3.3. Intestinal Tissue Sampling and Analyses

At the end of the experiment, a total of eight animals from each experimental group were slaughtered. The intestinal tract was removed for histologic measurements analysis.

Segments of approximately 3 cm were collected from the duodenum and ileum and fixed in 4% (*v*/*v*) phosphate-buffered-saline paraformaldehyde for 24 h at 4 °C. The samples were then embedded in paraffin wax. Serial Sections (5 μm thick) were cut and stained with haematoxylin-eosin for morphological studies and by conventional histochemical procedures for the count of goblet cells (GCs) and for the characterisation of mucins.

The presence of goblet cells (GCs) and their contained glycans were demonstrated with (1) the periodic acid-Schiff (PAS) reaction for neutral glycans [46]; and (2) combined high iron diamine (HID)/Alcian Blue pH 2.5 (AB 2.5) for simultaneous staining of sulphated and non-sulphated acidic glycans [47].

### 2.4. Statistical Analysis

The data on the oxidative activity assays of leaves were analysed by one-way analysis of variance (ANOVA) and Tukey post hoc test using SPSS software for Windows version 23.0 (Inc., Chicago, IL, USA). Statistical significance was accepted at a level of *p* < 0.05. The results are expressed as mean values and standard deviations. Data collected from the biochemical, immunological and oxidative status of the animals were analysed using statistical SPSS software for Windows version 23.0 (Inc., Chicago, IL, USA). The difference between the means of the experimental groups was carried out using a one-way analysis of variance (ANOVA). Differences were considered statistically significant at *p* < 0.05.

Data on morphometric measurements were evaluated for statistical significance. Specifically, Haemoxylin-eosin-stained sections of 10 well-oriented villi and crypts of duodenum and ileum from each animal were photographed with a 4x lens using a light microscope (Eclipse Ni-U; Nikon, Japan) and used to measure the villus height (VH) and the crypt depth (CD). The density of GCs per 100 μm of villus length, as well as the number of GCs with different types of mucins as distinguished by PAS staining and the HID/AB 2.5 procedure, was also determined by counting the cell number per 20 randomly photographed fields of well-oriented villi of duodenum and ileum from each animal [48]. The photos were taken with a 60x lens. Data were evaluated for statistical significance by Student’s t-test; *p* value < 0.05 was considered significant. The values were expressed as mean ± standard deviation (S.D.).

## 3. Results

### 3.1. In Vitro Assays

#### Chemical-Based Antioxidant and Interaction Activity of the Leafs Mixture

The results on the antioxidant activities, estimated by TPC, ORAC, FRAP, and TEAC-ABTS, are reported for individual leaves (OL, BL, RL) and their combinations (OL + BL, OL + RL, BL + RL, OL + BL + RL) in Table 2. Among the individual leaves, OL showed the highest (*p* < 0.05) TPC value in comparison with BL and RL (*p* < 0.05). The high TPC of OL influenced the phenol content of binary leaves combination, which resulted higher in those where OL was included (OL + BL, and OL + RL); BL + RL had the lowest TPC value that resulted in significant differences (*p* < 0.05) compared to OL + BL. The ternary leaf combination resulted in the highest TPC value when compared to both individual BL (*p* < 0.05) and RL, and in comparison, with their binary mixture (BL + RL; *p* < 0.05). No differences (*p* > 0.05) were observed between the binary combinations with the inclusion of olive leaves (OL + BL, and OL + RL) (Table 2).

The ORAC assay exhibited the highest antioxidant capacity of OL as individual leaves compared to BL and RL (*p* < 0.05), as well as in its binary combinations OL + BL and OL + RL compared to BL + RL (*p* < 0.05) (Table 2). The ORAC value of the ternary combination was 1048 ± 10.56 μM TE g^−1^, with a significant difference in comparison to the BL + RL combination (*p* < 0.05) (Table 2).

The antioxidant capacity estimated by FRAP assay (Table 2) showed the highest (*p* < 0.01) value for BL single leaves. The positive antioxidant effect of BL was decreased in all mixtures in which it was included. The ternary combination of the leaves (OL + BL + RL) improved the FRAP value in comparison to the binary leaf combinations (OL + BL and OL + RL; *p* < 0.05).

No differences (*p* > 0.05) were found for TEAC-ABTS assay among the individual leaves and their combinations, with the values ranging from 36.49 to 59.05 μM TE g^−1^ (BL + RL and OL + BL + RL, respectively) (Table 2).

Table 3 illustrates the results of the synergistic action of the binary and ternary mixtures of the leaves. The fa (affected fractions)-CI values of the OL, BL, and RL combinations at 50%, 75% and 90% antioxidant effect levels, indicate that the ternary LPM (OL + BL + RL) possesses the synergistic effect (CI, 0.60–0.63), compared to the binary combinations which exhibited moderate synergism (OL + BL: CI, 0.70–0.80) or antagonism activity (OL + OR: CI, 1.17–2.27; BL + OR: CI, 1.25–1.58).

The ternary LPM at the fraction affected (fa) of 50% obtained by CompuSyn software 1.0., indicated a combination satisfying for LPM, corresponding for OL to 65.7%, for RL to 18.9%, and for BL to 15.4%. These ratios have been used in the in vivo study for the formulation of LPM supplementation, as weight proportions of the leaves, in the diet of laying hens.

### 3.2. In Vivo Study

#### 3.2.1. Biochemical Parameters

The effects of dietary supplementation of LPM on blood biochemical parameters, lipid and immunological profiles, and liver activity in laying hens at the end of the experiment are presented in Table 4. Biochemical blood constituents revealed significant differences between dietary treatments. The hens treated with LPM had a significant decrease (*p* < 0.05) in triglycerides, total cholesterol, and LDL cholesterol and increased levels (*p* < 0.05) of HDL cholesterol, compared to the control group.

The immunomodulatory effects reported in Table 4 indicate that the mean values of IL-1β and IL-6 were significantly decreased in hens treated with LPM when compared with the control group (*p* < 0.05).

The means of ALT, AST, and ALP serum content showed differences between groups (Table 4). In hens supplemented with LPM, a significant decrease (*p* < 0.05) in ALT and AST activity was observed compared to the control group. There were no significant differences (*p* > 0.05) between groups in ALP levels.

Table 5 shows the effects of dietary LPM supplementation on the oxidative status of hens. The LPM group showed higher levels (*p* < 0.05) of TAS and FRAP test values than the control group. Plasma levels of ROMs and TBARs were significantly lower (*p* < 0.05) in the hens treated with LPM.

The LPM-treated hens also showed higher levels (*p* < 0.05) of vitamin E than the control group.

#### 3.2.2. Histological Morphometry and Histochemistry Analyses

**Figure 1:**
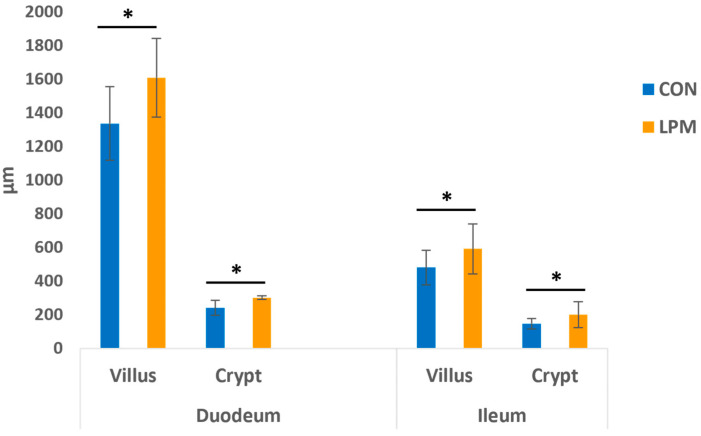
Effect of dietary leaf power mixture (LPM) supplementation on laying hens’ villus height and crypt depth. Data show the mean with error bars representing ± SD and Student’s *t*-test results. * *p* < 0.05.

The examined tissues from CON and LPM samples did not show either macroscopic or histological lesions. The intestinal mucosa consisted of villi and basal crypts both covered by a simple columnar epithelium and scattered GCs. The morphometric evaluations revealed that the LPM significantly increased the villus height and the crypt depth of the duodenum and ileum (Figure 1). No statistical difference was observed in the VH: CD ratio between the control and LPM-treated groups, being 5.5 vs. 5.3 in the duodenum and 3.25 vs. 2.95 in the ileum.

**Figure 2:**
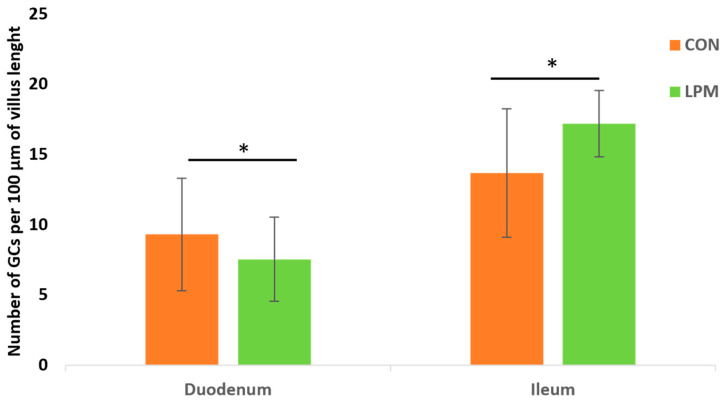
Density of goblet cells (GCs) expressed as the number of cells per 100 µm of villus length in duodenum and ileum of control (Con) and leaf power mixture (LPM) supplemented laying hens stained with PAS and HID/AB 2.5 procedure to reveal both neutral and acidic mucins. Data show the mean with error bars representing ± SD and Student’s t-test results. * *p* < 0.05.

PAS and HID/AB 2.5 sequential staining showed that the density of GCs (goblet cell numbers per 100 μm of villus length) was lower significantly (*p* < 0.001) in the duodenum than ileum in both the CON and LPM avians (Figure 2). The LPM administration reduced (*p* < 0.05) the density of GCs in the duodenum, whereas it significantly (*p* < 0.001) increased the density of GCs in the ileum (Figure 2).

**Figure 3:**
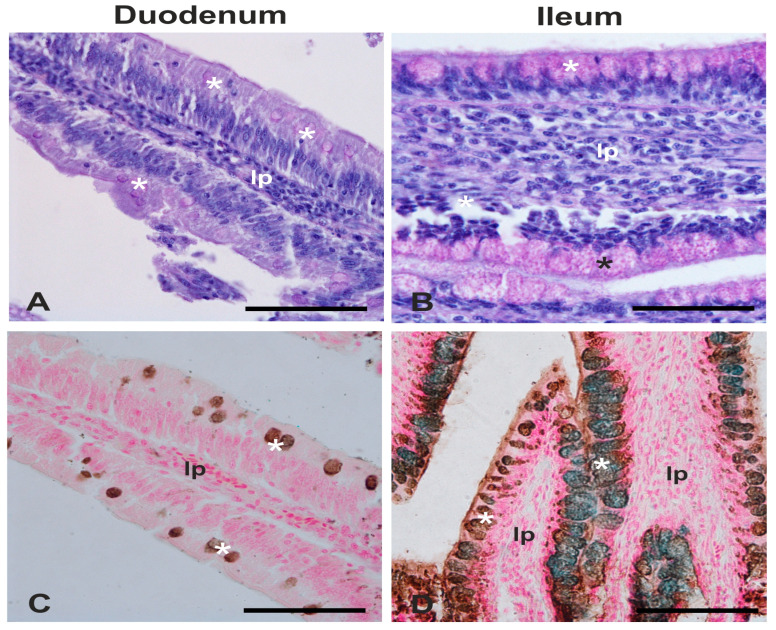
Representative pictures showing the different staining intensity with PAS (**A**,**B**) and HID/Alcian Blue pH 2.5 (AB2.5) (**C**,**D**) staining procedures in the duodenum and ileum villi of laying hens. (**A**,**B**) PAS-positive goblet cells exhibit magenta staining; the nuclei are stained with Mayer’s hemalum. (**C**,**D**) Goblet cells show HID positivity (brown) in the duodenum and both HID and AB 2.5 positivity (blue) in the ileum; the nuclei are stained with fast red. lp, lamina propria; asterisk, goblet cells. Scale bar: (**A**–**D**), 25 µm.

**Figure 4:**
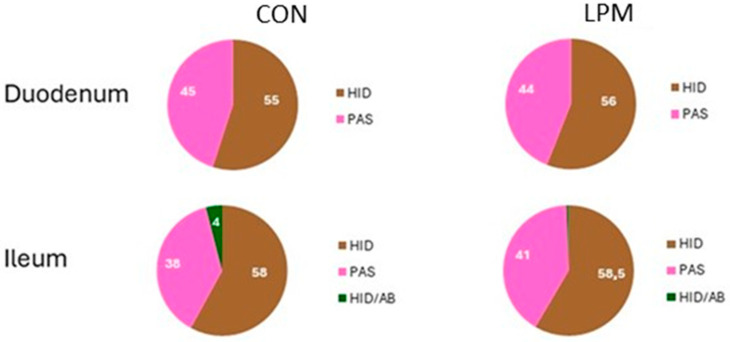
Percentage of the intestinal goblet cells of Control and LPM hens producing neutral mucins (PAS, magenta), acidic sulphated glycans (HID, brown), and both non-sulphated and sulphated acidic glycans (HID/AB 2.5, green).

Generally, the intensity of PAS staining was weaker when compared to HID and AB2.5 stains (Figure 3) and the staining intensity of the duodenum was weaker than ileum. Regardless of the intestinal tract investigated, a predominance of GCs producing acidic glycans on the GCs producing neutral glycans in both Con and LPM hens was observed (Figure 3 and Figure 4).

In the duodenum, the acidic mucins were exclusively stained with HID procedure (Figure 4). The LPM treatment did not significantly modify the percentage of GCs producing the neutral and the acidic mucins in the duodenum (Figure 4). In the ileum, the percentage of acidic mucins producing GCs was higher than in the duodenum. The HID/AB 2.5 staining procedure revealed the presence of both HID and HID/AB2.5 positive GCs, the HID-positive GCs were much more numerous than HID/AB2.5 positive GCs. LPM treatment induced a low increase in the percentage of GCs, producing neutral glycans and a high reduction in HID/AB2.5 positive GCs.

## 4. Discussion

### 4.1. In Vitro Study

This study was conducted to assess the antioxidant potential of olive, bay, and rosemary leaves, as well as their interaction through in vitro testing and in vivo validation in laying hens. This research focused on evaluating the antioxidant activity of OL, BL, and RL powders and identifying possible interactions between them. For a more accurate evaluation of the antioxidant activity, various assessment testing methods were employed, following the approach of Verhagen et al. [30].

OL exhibited greater TPC and antioxidant capacity than BL and RL, and binary combinations including OL showed a higher antioxidant capacity. The higher antioxidant activity of OL compared to BL and RL aligns with findings from other studies [49,50,51]. A positive correlation between TPC and the antioxidant capacity of foods or herbal has been previously reported [34,52,53], consistent with our results. Antioxidant activity is ascribed to the ability of polyphenols to donate hydrogen molecules, quenching singlet oxygen, acting as chelators and trapping free radicals [1,2].

Different results in terms of antioxidant capacities were observed in this study for the ORAC, FRAP, and TEAC-ABTS tests. Variability is common in the evaluation of antioxidant capacities in relation to the test used and the reaction mechanisms involved, the combination of bioactive components, and their structure [54,55]. It should be noted that the antioxidant effectiveness of most polyphenols is based on hydrogen atoms and electron transfer [56], with the ORAC test focusing on the former and the FRAP and ABTS tests on the latter. The Ternary combination of LPM showed the highest antioxidant capability, with significant differences in TPC and ORAC tests; therefore, ORAC values were selected for the synergistic antioxidant evaluation using the CI method.

The advantages of combining drugs or phytochemicals dietary additives are well known due to an improved understanding of biological systems [22,26,57,58]. The synergistic model proposes that a combination of drug and/or bioactive phytochemicals combination produces greater effects than the individual components can achieve alone [59,60]. Combined treatments rely on compounds that interact with different molecules or pathways, offering various advantages [31,61], such as targeting dysregulated signalling [62]. Phytochemicals dietary additive or chemotherapy approaches are often built around synergistic interactions [59,63].

In the present study, the binary combination of olive and bay (OL + BL) leaves exhibited additive or moderate synergism, while the binary combinations including rosemary (OL + OR and BL + RL) exhibited antagonism effects. It has also been observed that phytochemicals [64] and food categories [25] combined in pairs can result in either synergistic or antagonistic effects. According to Sazhina, [65], the additive effect is the cause of the absence of synergistic or antagonistic interactions between bioactive components; in such cases, the bioactive component in the complex mixture acts independently of each other. Furthermore, the inhibition of active biocompounds by other co-existing compounds may lead to antagonistic effects. Again, the antagonistic or synergistic, interaction can also be explained through a regeneration mechanism: where a more effective antioxidant is replaced by a less effective one, or synergy occurs when a less effective antioxidant regenerates the more effective one [66,67,68].

In this study, the CI values for the ternary LPM indicate the absence of antagonism among OL + OR + BL. Olszowy-Tomczyk, [69] reported that the synergistic antioxidant activity can be justified in complex mixtures of bioactive components, based on the formation of stronger antioxidants from weaker antioxidants, highly stable intermolecular complexes, and/or new phenolic compounds with high antioxidant activity compared to their precursor components. In the present study, the ternary LPM combinations (OL + OR + BL) showed a lower CI value (0.63), indicating synergistic effects compared to the other combinations. Thus, the ternary LPM combinations were considered the strongest candidate for in vivo validation of antioxidant synergism.

### 4.2. In Vivo Study

Dietary polyphenols can exert an effect on biological systems through various mechanisms. In this study, dietary supplementation with the ternary LPM (OL + BL + RL) led to a decrease in total cholesterol, triglycerides, and LDLc and an increase in HDLc in hens. Regarding the effects of dietary phytochemical natural additive, olive leaf supplementation caused a decrease in blood cholesterol levels in Japanese quails [70] and in broilers [71]. It has been reported that oleuropein and hydroxytyrosol, found in olive leaves, inhibit 3-hydroxy-3-methyglutaryl coenzyme A that plays an important role in cholesterol synthesis in liver [15], and phenolic compounds of olive leaves may inhibit cholesterol absorption in the intestine or its production by the liver or stimulation of the biliary secretion of cholesterol and cholesterol excretion in the faeces [72]. Torki et al. [73] reported that adding essential oil of rosemary to the diet of laying hens decreased the serum concentration of triglyceride and cholesterol. Ali and AL-Shuhaib [74] showed that adding crushed laurel leaves (2–3 g/kg of feed) to the diet led to increased HDL concentration and decreased TC, TG, and LDL in broilers. Fruchart et al., [75] reported that, in lipid metabolism, polyphenols activate the peroxisome proliferator-activated receptor (PPAR)-α, modulating the expression of key proteins in the liver involved in HDLc metabolism. The same mechanism promotes lipoprotein lipase in peripheral tissues and an increase in lipolysis, which results in reduced levels of circulating triglycerides and very low-density lipoproteins.

Oxidant/antioxidant markers are indicative of the physiological status of animals. In the present study, the improvement in oxidative status of hens treated with ternary synergistic LPM could be attributed to their major polyphenol constituents, mainly to oleuropein and its derivative hydroxytyrosol, for olive leaves [13] to 1.8 cineole, for laurel leaves [76,77], and to the antioxidant properties of carnosol and carnosic acid, for rosemary [78]. The lower ROM and TBAR levels observed in treated hens express the capacity of LPM to reduce free radical production and oxidative stress and are associated with higher TAS and FRAP levels. Most phenolic compounds act by either donating hydrogen or electrons, by breaking the free radical chain reaction, by preventing the chelation of metal ions, or by inhibiting the starting of the oxidative process through pro-oxidant enzyme downregulation [79,80]. Furthermore, polyphenols contribute to the stabilisation of low molecular weight molecules, such as vitamin E, by increasing plasma antioxidant capacity [81,82]. Our results were consistent with findings related to single foliar treatment. Sarica et al., [83] reported that oleuropein supplementation increased TAS in quails. Rosemary and laurel leaves were shown to inhibit TBARs [51] and can protect the brain from the damage of free radicals [84]. TAS increased with the dietary integration of rosemary or laurel leaves in lambs [85] and rabbits [86]. In addition, mixed proportions of herbs and plants can affect antioxidant properties [87] because the total antioxidant capacity is ascribed to their additive synergistic effects [9], in accordance with the results of this study.

Immunological effects of phytochemical dietary additives play an important role in counteracting oxidative mechanisms. Oxidative stress represents a common final pathway related to inflammation, though the molecular mechanisms of the interaction between oxidative stress and inflammation remain unclear [88]. Adipokines are involved in the regulation of several physiological processes [89], including the control of oxidative stress [90]. The findings of this study indicated that LPM of OL + RL + BL showed anti-inflammatory and immunomodulatory properties, as evidenced by a significant reduction in inflammatory mediators such as IL-1β and IL-6. Miliaraki et al. [91] reported a strong correlation between the cytokines TNF-α and IL-6 with TAC, which aligns with our results. Our results are in line with studies on the use of individual plants [92]. The immune system benefits from olive leaf extracts [93,94], bay leaves [95], and rosemary leaf powder [96].

A marked improvement was observed in liver function enzymatic parameters in the experimental groups integrated LPM. Hepatic injuries lead to the attenuation of metabolic functions regulated by the liver. The evaluation of liver enzymatic activities is a valuable tool for determining the safe inclusion rate of non-conventional feed or feed additives in poultry [97,98]. The finding of this study demonstrated that LPM has hepatoprotective effects consistent with the results of many studies where a decrease in AST, ALT, and ALP levels is associated with the antioxidant activity of phytochemicals in plant leaves or extracts. Mohammed et al., [99] reported the hepatoprotective effects of laurel leaves in rats, while Al-Attar and Shawush [100] demonstrated that the extracts of olive and rosemary leaves possess hepatoprotective properties against liver cirrhosis induced by thioacetamide by inhibiting the physiological and histopathological alterations attributed to their antioxidant activities. Abdel-Azeem et al. [101] reported that laurel leaf supplementation in rabbits’ diets showed a significant decrease in AST and ALT blood plasma, in agreement with our results.

In summary, synergism can occur through several mechanisms, involving different molecules or pathways that enhance antioxidant activity and increase potency [25,102], as well as improve metabolic profile and intestinal function [25,103,104], which aligns with the results observed in this study.

### 4.3. Intestinal Morpho-Histochemical Characteristics

Histomorphometry analysis revealed that LPM induces a significant increase in the VH of the duodenum and the ileum of laying hens. This increase in intestinal VH has been related to the increase in the effective absorptive surface area by increasing brush border enzymes involved in facilitating digestion, improving the nutrient transport system and absorption of available nutrients [105,106,107]. Thus, although the digestibility assay was not examined in this study, these findings suggest that the administered LPM exerts beneficial effects on the absorptive capacity of the intestinal mucosa.

LPM supplementation significantly increased the CD of the duodenum and the ileum. It has been reported that CD is directly representative of the intestinal environment and may be used to assess intestinal health [108]. Therefore, these CD findings support the increase in VH, as intestinal crypts consist of proliferating cell types that differentiate into villus epithelial cells. Villus height and crypt depth represent an indirect indication of the maturity and functional capacity of enterocytes. Longer villi and deeper crypts indicate a greater number of enterocytes [109]. This could justify the unchanged VH:CD ratio in the duodenum and ileum of the control and LPM-treated groups.

GSs produce the mucins constituting the mucus layer that covers the luminal surface of the epithelium. The mucus layer serves as the physical barrier which prevents invasion of the intestinal epithelial cells by gut pathogenic bacteria and viruses, while selectively facilitating adherent growth of normal resident gut microbiota [110]. The density of GCs (cell numbers/100 μm of villus length) was lower in the duodenum than the ileum in both the CON and LPM groups. A progressive increase in goblet cells density along the duodenal–ileal axis has been found in other avian species [111,112]. Although LPM administration induced an increase in the VH and CD of the duodenum and ileum, histochemical results did not evidence a simultaneous increase in the GC density in the two examined intestinal tracts. Specifically, the LPM group displayed a decrease in GS density in the duodenum and an increase in GC density in the ileum. This regional opposite effect is challenging to interpret. Probably, it could depend on the different roles of the duodenum and ileum, because the duodenum also showed a lower content of mucins (mucin staining intensity) when compared to ileum. A decrease in mucin staining intensity between duodenum and ileum has been reported in broiler chickens [111]. Since the distal ileum is a potential preferred region for bacterial colonisation [113], it has been suggested that the microbial dynamics occurring in the ileum could induce a higher production of mucins to ensure greater protection [111].

Mucins can be classified into neutral and acidic types, with the latter further categorised as sialomucins and sulfomucins. Histochemical results demonstrated that GCs produced mainly acidic mucins in the duodenum and ileum, although a greater presence of both neutral and acidic mucins was observed in the ileum than in the duodenum. A scarce presence of GCs containing neutral mucins has also been observed in the intestinal villi of the small intestine of other avians [112,114,115,116]. In the present study, LPM treatment did not affect the number of GCs producing the neutral and the acidic mucins in the duodenum, where the acid glycans were only sulphate type. The function of neutral mucins is not fully understood. This type of mucin, together with the pancreatic juice, is involved in the neutralisation of the acidic pH of gastric juices entering the duodenum [117]. As for the acidic mucins, several studies evidenced that the role of this type of glycan is to protect the gut wall against bacterial glycosidase and proteases [118,119,120]. This protective activity may be attributed to the increased overall negative charge of acidic mucins [121,122,123].

In the ileum of CON group, the number of GCs containing neutral mucins was lower compared to the duodenum, and the acidic mucin secretory GCs were mainly constituted of sulphated mucin GCs, with only 4% of the GCs containing non-sulphated mucins (sialomucins). The presence of non-sulphated glycans may indicate the presence of incompletely matured acidic glycans, as sulphated glycans are in most cases extensions of the sialylated glycans [120]. The increased presence of acidic mucins in the ileum could depend on the high bacterial colonisation occurring in this intestinal tract [113] and the need for increased protection, resulting in increased production of acid mucins [111]. LPM supplementation induced a low increase in the number of GCs containing neutral glycans at the expense of the acid mucin-producing GCs, which predominantly contained sulphated mucins. The increase in GCs producing the neutral mucins in LPM-supplemented hens could depend on the protective effect of LPM by impeding colonisation by enteric pathogens and reducing gastrointestinal infections. It has been reported that neutral mucins could play a role in protection against invasion by pathogenic bacteria [124].

Ultimately, the results indicated that dietary LPM improved intestinal function in hens by protecting their intestinal structure and integrity. LPM exerted synergistic effects on intestinal morphology by promoting villi height crypt depth, and CG activity.

## 5. Conclusions

This is one of the few studies where LPM (OL + BL + RL) was evaluated through in vitro chemical tests and validated in an in vivo study.

The in vitro study indicated a higher antioxidant capacity of olive leaves, and combining olive leaves with laurel or rosemary enhances the antioxidant capacity. The ternary LPM showed stronger antioxidant capability than the respective single/double-agent treatments in vitro tests. Its CI value at IC 50 concentration (0.60) indicated the synergistic activity of LPM. The beneficial effect of ternary LPM (OL + BL + RL) was confirmed in the in vivo study, which showed improved oxidative status (TAS, FRAP; ROMs, TBARs), and vitamin E level, enhanced metabolic profile (lipidic, hepatic, immunological) as well as improved intestinal morpho-histochemical characteristics (villi height, crypt depth, CGs feature) in laying hens. The synergistic antioxidant results of LPM (OL, RL, BL) can provide a theoretical basis for the development of pathways for the formulas of functional food ingredients. Moreover, this study may contribute to the development of promising, low cost and applicable dietary combination with enhanced antioxidant properties for laying hens.

## Figures and Tables

**Table 1:** Composition and nutrient levels of the control diet (%).

Ingredients	%
Corn	57.60
Soyabean meal (46% CP)	22.00
Sunflower flour (36% CP)	6.00
Limestone granular	6.00
Limestone	3.30
Soyabean oil	2.50
Dicalcium phosphate	1.50
Vitamin and mineral premix ^1^	0.50
Sodium chloride	0.20
Sodium bicarbonate	0.15
Methionine (MHA) ^2^	0.14
Lysine	0.09
Magnesium oxide	0.02
**Nutrient levels** ^3^	
Metabolizable energy (kcal/kg)	2700
Crude protein	17.80
Crude fat	4.40
Calcium	4.10
Phosphorus (available)	0.45
Methionine	0.31
Lysine	0.74
Arginine	0.70
Threonine	0.37
Leucine	0.74
Isoleucine	0.43
Valine	0.46
Histidine	0.25
Phenylalanine	0.48
Tryptophan	0.13

^1^ Supplied per kilogramme of diet: vitamin A, 10,000 IU; vitamin D_3_, 3000 IU; biotin, 0.08 mg; choline chloride, 350.00 mg; folic acid, 0.80 mg; niacin, 29.50 mg; vitamin B_1_, 1.96 mg; vitamin B_12_, 0.02 mg; vitamin B_2_, 4.00 mg; vitamin B_6_, 3.96 mg; vitamin E, 25.00 mg; vitamin K_3_, 1.79 mg; enzymes: endo-1,4-betaglucanasi; endo-1,4-betaxilanasi; 3-fitasi; iron, 48.00 mg; iodine, 1.02 mg; manganese, 80.60 mg; calcium D-pantothenate, 8.91 mg; selenium, 0.15 mg; zinc, 79.2 mg; copper, 12.50 mg. ^2^ Methionine hydroxy analogue. ^3^ Calculated using NRC (1994) values.

**Table 2:** Total phenolic content and antioxidant capacity of leaves of olive (*Olea europaea* L.), rosemary (*Rosmarinus officinalis*) and laurel (*Laurus nobilis* L.) through various mechanisms of antioxidant action.

Sample	TPC (gGAE/kg) ^1^	ORAC (TE μM/g) ^1^	FRAP (TE μM/g) ^1^	TEAC-ABTS (TE μM/g) ^1^
	x ± SD	x ± SD	x ± SD	x ± SD
*Significant level*	**	**	**	n.s.
OL	6.58 ± 0.48 ^a^	1035.95 ± 21.99 ^a^	61.82 ± 13.03 ^a^	47.31 ± 12.45
BL	4.89 ± 0.68 ^b^	895.12 ± 10.65 ^b^	114.60 ± 14.10 ^b^	44.03 ± 10.80
RL	5.10 ± 0.71 ^b^	938.39 ± 42.93 ^b^	56.01 ± 4.64 ^a^	44.16 ± 5.30
OL + BL	5.41 ± 0.37 ^a^	1016.70 ± 24.41 ^a^	30.34 ± 7.08 ^b^	43.84 ± 7.31
OL + RL	6.60 ± 0.22 ^a^	1041.35 ± 20.33 ^a^	32.65 ± 6.57 ^b^	50.28 ± 10.61
BL + RL	4.44 ±0.04 ^b^	846.29 ± 11.91 ^b^	58.54 ± 14.97 ^a^	36.49 ± 5.00
OL + BL + RL	6.62 ± 0.31 ^a^	1048.71 ± 10.56 ^a^	64.85 ± 15.51 ^a^	59.05 ±8.58

OL: olive leaf; BL: bay laurel leaf; RL: rosemary leaf. ^1^ Total phenolic content (TPC) results (n. 3) are expressed as g gallic acid equivalent (GAE) in kg of dry weight (dw); ORAC and FRAP (n. 4) results are expressed as micromole Trolox equivalents (TEs) in grammes dw; TEAC-ABTS (n. 6) results are expressed as millimole TE in grammes dw. ** = *p* < 0.01; n.s. = not significant. ^a,b^ Means within columns with different superscript letters are significantly different for *p* = 0.05, according to the Tukey post hoc test.

**Table 3:** Potential synergistic antioxidant interaction of olive (OL), bay (BL) and rosemary (RL) leaves in binary and ternary combinations.

Leaf Mixture	fa	CI Value→Interpretation
OL + BL	0.50	0.926→ADDITIVE
	0.75	0.804→Moderate SYNERGISM
	0.90	0.702→Moderate SYNERGISM
OL + RL	0.50	1.171→Slight ANTAGONISM
	0.75	1.576→Moderate strong ANTAGONISM
	0.90	2.277→Moderate strong ANTAGONISM
BL + RL	0.50	1.248→ANTAGONISM
	0.75	1.405→ANTAGONISM
	0.90	1.585→Moderate strong ANTAGONISM
OL + BL + RL	0.50	0.630→SYNERGISM
	0.75	0.616→SYNERGISM
	0.90	0.601→SYNERGISM

fa = fraction affected; CI = combination index. CI values of each leaf mixture are presented at 50%, 75% and 90% of fa. The combination effect (synergism, additivity, and antagonism) was interpreted based on CI values: <0.3 strong synergism, 0.3–0.69 synergism, 0.70–0.84 moderate synergism, 0.85–0.89 mild synergism, 0.9–1.09 additive, 1.10–1.19 slight antagonism, 1.20–1.44 antagonism, and >1.45 moderate to strong antagonism [39].

**Table 4:** Blood lipidic, hepatic and immunological profiles in laying hens supplemented with dietary LPM.

	Dietary Treatment		SEM	*p* Value
	CON	LPM		
Animals, n.	30	30		
Triglycerides, mg/dL	831.25	702.41	43.90	0.049
Total cholesterol, mg/dL	139.20	124.60	1.24	0.046
HDL cholesterol, mmol/L	20.66	28.62	0.57	0.032
LDL cholesterol, mg/dL	141.44	135.15	1.28	0.037
AST, IU/L	184.23	172.21	2.71	0.048
ALT, IU/L	16.39	13.04	0.471	0.041
ALP, IU/L	282.54	264.38	15.08	0.370
TNF-α, pg/mL	29.35	25.70	1.12	0.650
IL-1β, pg/mL	64.41	52.63	2.47	0.022
IL-6, pg/mL	20.19	16.95	1.09	0.041

CON, control basal diet; LPM, diet supplemented with leaf powder mixture, 6 g/kg feed; SEM: standard error mean. HDL = high-density lipoprotein; LDL = low-density lipoprotein. AST = aspartate aminotransferase; ALT = alanine aminotransferase enzyme; ALP = alkaline phosphatase; TNF-α = tumour necrosis factor; IL-1α = interleukine-1β; IL-6 = interleukine-6.

**Table 5:** Serum oxidative status markers and levels of vitamins in laying hens supplemented with dietary LPM.

	Dietary Treatment		SEM	*p* Value
	CON	LPM		
Animals, n.	30	30		
TAS (Trolox Eq/L)	354.18	407.15	38.84	0.029
FRAP (TE μM/g)	315.19	346.67	35.16	0.046
ROMs (UCarr)	39.70	21.18	4.81	0.049
TBARs (μmol/L)	26.91	20.54	2.78	0.050
Vitamin A (μg/mL)	1.123	1.160	0.038	0.284
Vitamin E (μg/mL)	1.581	1.854	0.046	0.042

CON, control basal diet; LPM, diet supplemented with leaf powder mixture, 6 g/kg feed; SEM: standard error mean; TAS = total antioxidant status; FRAP = ferric reducing antioxidant power; ROMs = reactive oxygen metabolites; TBARs = thiobarbituric acid reactive substances.

## Data Availability

Data are contained within the article.
